# Mobilising people as assets for active ageing promotion: a multi-stakeholder perspective on peer volunteering initiatives

**DOI:** 10.1186/s12889-020-10136-2

**Published:** 2021-01-18

**Authors:** Afroditi Stathi, Janet Withall, Sandra Agyapong-Badu, Eva Barrett, Marlene Kritz, Debbie Wills, Cecilie Thogersen-Ntoumani, Kenneth R. Fox

**Affiliations:** 1 School of Sport, Exercise and Rehabilitation Sciences, University of Birmingham, Birmingham, UK; 2 Department for Health, University of Bath, Bath, UK; 3 School of Nursing and Midwifery, National University of Ireland, Galway, Ireland; 4 School of Psychology, Curtin University, Perth, Australia; 5 St. Monica Trust, Bristol, UK; 6 School of Policy Studies, University of Bristol, Bristol, UK

**Keywords:** Volunteering, Physical activity, Qualitative synthesis, Health promotion, Community

## Abstract

**Background:**

Successful peer volunteering is central to many community-based, active ageing initiatives. This study synthesises the perspectives of a range of stakeholders involved in peer volunteering initiatives and provides recommendations as to how peer volunteers can be effectively mobilised as community assets.

**Methods:**

An evidence synthesis of qualitative data from (a) the evaluation of ACE (Active, Connected, Engaged), a feasibility trial of a peer volunteering active ageing intervention, and (b) interviews with volunteers and managers of third sector organisations providing peer volunteering programmes. Data were analysed using directed content analysis.

**Results:**

Ten managers, 22 volunteers and 20 ACE participants were interviewed. The analysis identified six main themes, 33 higher and 22 sub themes. Main themes were: (i) Motives, (ii) Benefits, (iii) Skills and Characteristics, (iv) Challenges, (v) Training Needs, (vi) Recruitment and Retention. Altruism, changes in life circumstances, opportunities to reconnect with the community and personal fulfilment were the main reasons for volunteering. Volunteering was described as being personally rewarding, an avenue to acquire new skills and knowledge, and an opportunity for increased social connections and physical activity. Good peer volunteers are committed, reliable, have a good sense of humour, good interpersonal skills and are able to relate to participants. When pairing volunteers with participants, shared interests and geographical proximity are important to consider. Clarity of role, level of time commitment, regular feedback, recognition of effort and strong networks for on-going support are important strategies to facilitate volunteer retention.

**Conclusions:**

The findings of this study support the value of peer volunteering as a strategy for mobilising community assets in promoting active ageing. To ensure success and longevity, these schemes require appropriate funding and efficient administrative support.

**Supplementary Information:**

The online version contains supplementary material available at 10.1186/s12889-020-10136-2.

## Background

Prioritising the mobilisation of community assets is at the centre of public health promotion in the United Kingdom [UK] [1, 2]. In England, the National Institute for Health and Care Excellence identifies five broad categories of assets that can support health and wellbeing including skills, knowledge and commitment of individual community members; friendships, community cohesion and neighbourliness within a community; local groups and community and voluntary associations; physical, environmental and economic resources within a community; and assets brought by external agencies [3].

Central to the impact of a community asset approach is the engagement of communities and individuals within those communities, in decision-making regarding local initiatives and their execution. This is strongly supported in government health promotion and self-care policies [4, 5], particularly as it may be a valuable contributor to tackling ever-increasing health inequalities, an issue starkly illustrated by the 19-year gap in healthy life expectancy between those living in England’s most and least deprived areas and similar data from the United States of America [USA] [6, 7].

Local government and voluntary groups have a vital role in building the confident and connected communities that are central to a community asset approach. In such communities, all groups, but especially those at highest health risk, can tap into existing sources of social support and social networks, have a voice in shaping services and are able to play an active part in community life [8–10]. Building confident communities will be crucial in the post COVID-19 era where the return to ‘normality’ will be challenging, especially for older adults and those classified as vulnerable who have been isolated for several months.

In tackling this challenge, mobilisation of older adults as community assets to promote active ageing and reduce loneliness and social isolation, has received significant attention [10–13]. Citizens who contribute as volunteers can help by providing advice and assistance, or by organising and supporting engagement with activities to enhance health and wellbeing [14]. Peers can also be an excellent source of motivation and social support for older adults [15–17]. From a theoretical perspective, both social cognitive theory [18] and self-determination theory [19] suggest that peer volunteers can enhance the self-efficacy of participants through modelling (e.g., seeing others cope with barriers to active ageing), verbal encouragement, reduction in perceived barriers and increase levels of social support and connectedness. Volunteers themselves also benefit by experiencing higher levels of competence and relatedness [20–26].

Older people often face specific age-related barriers to volunteering, or experience life circumstances (personal ill health, being a carer for a significant other) that force them to withdraw from a volunteer role. It is therefore important to fully understand their experiences and support them in facing these personal challenges and those associated with the volunteering role. These important issues have not yet been comprehensively addressed in the literature [27].

The aim of this study was to explore a) the importance of peer volunteering and b) identify ways for improving the process of mobilising older adults as community assets in peer volunteering active ageing initiatives. The study draws on the experience of a range of stakeholders, including older volunteers, recipients of volunteering actions, volunteer managers and volunteering service providers. It identifies strategies to engage and retain older volunteers and provides guidance for enhancing the effectiveness of peer volunteering schemes.

## Methods

Design, data collection, and analysis are presented according to the consolidated criteria for reporting qualitative research (COREQ) [28].

### Design

The Active, Connected, Engaged [ACE] study was a theory-informed, feasibility study which tested the use of peer volunteering support to promote active ageing in socially disengaged, physically inactive older adults [16, 29]. In summary, 54 older adults were recruited into the ACE study as either peer volunteers (Activators; *n*=15) or participants (ACEs; *n*=39).

Participants were recruited via a letter of invitation. A commercial mailing list of residents in the relevant postcodes who were 65 and older was purchased and invitation letters and reply slips were mailed. Posters were displayed in local health centres, libraries, and community centres, and local community groups and professionals were asked to refer potential participants. People interested in taking part were sent an information pack, a reply form requesting basic demographic information, and a reply-paid envelope. Once screened and deemed eligible, ACEs were randomized to either the intervention or the control arm by choosing an envelope containing random allocation information. Using established communication channels and via the recruitment mailing described above, we recruited adults aged 60 and older to act as peer volunteers (see 20 for recruitment details). Activators were supported by paid volunteer coordinators (Coordinators; *n*=2). Participants were randomised to one-to-one support from an Activator or a waiting list, control group. The control group received written materials about local initiatives and were offered the intervention after study completion. For a six-month period, Activators supported the intervention group participants to get out and about more often and engage with local activities. Since the completion of the research study, the ACE programme has been delivered by a UK community organisation.

This research synthesis brings together qualitative data collected from a) volunteer managers and volunteers working with a range of community organisations b) volunteers (Activators) and participants in the ACE feasibility trial and c) volunteer managers in the community roll-out of the ACE programme.

All ACE participants lived or worked in the city of Bristol, United Kingdom. The University of Bath (EP 11/12 98) and the University of Birmingham (ERN_18–0877) Research Ethics Committees ethically approved the study. See detailed information on recruitment in [16, 29].

### Data collection

All study participants received a participant information sheet prior to recruitment. Written, informed consent was obtained before all group and one-to-one interviews took place. JW and AS, experienced qualitative researchers, conducted group and one-to-one interviews while training two novice researchers (IW and HT) who also conducted interviews under supervision. Two pilot group interviews, one with peer volunteers (*n*=2) and one with ACE participants (*n*=3) were conducted to further refine the interview guide and ensure that the language used was appropriate for all stakeholders. Group and one-to-one interviews were audio-taped using an Olympus VN2100PC digital voice recorder and transcribed verbatim. All transcripts were entered into NVivo Software for Qualitative Research 10 and coded ensuring anonymity and confidentiality.

We developed two interview guides, one for participants and one for peer volunteers and managers of volunteering organisations and initiatives [16, 29] which explored six topical areas: Motives for peer volunteering, Benefits of peer volunteering, Skills and characteristics of a good peer-volunteer, Peer volunteering challenges, Peer-volunteer training needs, Recruitment and retention of peer-volunteers.

### Data sources

The flexible approach offered to participants (semi-structured, one to one interviews or group interviews) was adopted in order to accommodate participants’ preferences and/or time availability. All interviews were conducted after the completion of the ACE intervention.

#### Data source a; managers of volunteering organisations and initiatives (*n*=10)

Semi-structured interviews were conducted with three ACE programme managers and three managers of volunteering organisations and a group interview (*n*=4) with managers from UK statutory and third sector organisations. The ACE programme managers were managing volunteers in either the ACE feasibility study (n=1) or in the ACE community programme (*n*=2). Other volunteer managers were identified through established communication channels with major local service providers.

#### Data source B; volunteers (*n*=22)

##### Interviews with experienced older adult peer volunteers (older volunteers, *n*=9)

Older volunteers were recruited via the community organisations for whom they volunteered. A range of peer volunteers working in various voluntary organisations were purposively selected to reflect variety in age, gender, and volunteering roles [30].

##### Group and one-to-one interviews with ACE peer volunteers (activators, *n*=13)

All 15 peer-volunteers (Activators) in the ACE feasibility trial were asked to participate in post-intervention focus groups or interviews. Twelve people took part in 4 group interviews and for logistical reasons one person attended an individual semi-structured interview. Two Activators were not interviewed, one had moved away and one was on an extended holiday. These were conducted in local community centres and lasted 40–60 min.

#### Data source C; ACE participants (*n*=20)

##### Group and one-to-one interviews with ACE intervention group participants (ACEs *n*=20)

All 39 participants in the ACE feasibility trial were asked to participate in post-intervention focus groups or interviews. Thirteen participants were interviewed in four group interviews and seven participants preferred individual interviews. The remaining 13 participants declined to be interviewed for either personal, health or logistical reasons. Both group interviews and individual interviews were conducted in local community centres or participants’ homes and lasted 40–60 min.

### Data analysis

Directed Content Analysis was used to interpret the data [31]. This is a systematic method of qualitative analysis, which uses both deductive and inductive reasoning. AS and JW started the analysis deductively, using the six thematic categories from the interview guide to develop the initial coding structure. This was then used to identify sub-themes in the data. The analysis then became inductive through searching for themes emerging from the data that did not fit or added new perspectives to the six thematic categories. To assure qualitative rigour, the researchers were responsive to emerging themes during data collection and these themes were discussed and agreed by AS, JW, SA and EB who analysed the data [32]. To minimise personal influences, the coding of the three data sources was shared among JW, SA and EB. AS reviewed, discussed and further refined the coding structure, which was approved by all authors. SA compared the results from the three data sources to identify similarities and differences. All authors discussed and agreed on the final analysis and interpretation of data [33].

## Results

### Participant characteristics

Ten female managers, 22 peer volunteers (including 13 ACE Activators) and 20 ACE participants (65% females; age range 67–94) were interviewed. The ACE Activators and participants’ characteristics are shown in Table 1.

**Table 1:** ACE Activators and participant characteristics

	Activators (*n*=13) n (%)	Participants (*n*=20)
Age (mean; years)	66.8 ± 4.3	75.9 ± 8.3
*Range*	63–74	67–94
Gender		
*Female*	10 (77)	13 (65)
*Male*	3 (23)	7 (35)
Ethnicity		
*White Caucasian*	13 (100)	19 (95)
*Other*		1 (5)
Marital status		
*Married*	7 (54)	6 (30)
*Widowed*	3 (23)	8 (40)
*Divorced/separated*	2 (15)	5 (25)
*Single/living with partner*	1 (8)	1 (5)
Highest education level completed		
*Completed secondary school*	5 (39)	14 (70)
*Some college/ vocational training*	1 (8)	3 (15)
*Completed college or university*	5 (39)	2 (10)
*Completed graduate degree or higher*	2 (15)	1 (5)

### Thematic structure

**Fig. 1:**
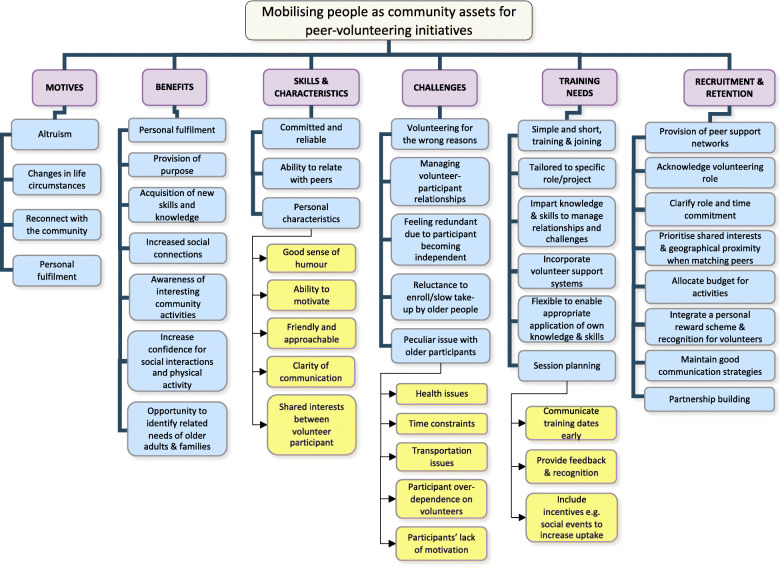
Thematic tree of key influences on mobilising people as community assets for peer-volunteering initiatives

Thirty-three higher and 13 lower order themes were identified within six main themes: Motives for peer volunteering; Benefits of peer volunteering; Characteristics of a good peer-volunteer; Peer volunteering challenges; Peer-volunteer training needs; Factors affecting recruitment and retention of peer-volunteers are presented in Fig. 1. Full thematic analysis is presented in Additional files 1, 2, 3, and 4.

### Motives, benefits of peer volunteering and characteristics of a good peer-volunteer

Altruism, changes in life circumstances, opportunities to reconnect with the community and personal fulfilment emerged as significant reasons for volunteering.

“I was re-housed up here. And I’d just taken early retirement so... I was looking for something to do. I came in to see what was going on..., I got well roped in” (Male, Older Volunteer- Data source B)

Volunteering was described as being personally rewarding and a route to acquiring new skills and knowledge. It provided a purpose in life, opportunities for social interactions and more involvement with community initiatives, including physical activity programmes.

“It’s nice having a new social circle; I’ve got to meet other people I wouldn’t have come across” (Male, ACE Activator, Data source B)

“For me it’s the reintegration into a social situation. It gives people a purpose. Once people are integrated … you know, you’re expected somewhere and wanted somewhere” (Female, Community initiative Manager, Data source A)

Participants stressed that important characteristics of a good peer-volunteer included a good sense of humour, commitment, reliability, good interpersonal skills, sharing common interests and the ability to relate to participants.

“I can honestly say never once since I met her, has she let me down” (Female, ACE Participant, Data source C)

“I found her easy to talk to and I didn’t know what to expect but I found her very friendly” (Female, ACE Participant, Data source C)

“The biggest thing I found was motivation. It was motivating people – to do things, trying things out, some work, some don’t” (Female, ACE Activator Data Source B)

Conversely, an apparent lack of commitment to the role had a negative impact on the participant and the delivery of the programme.

“And then she [peer volunteer] informed me she was away for x number of weeks and couldn’t see me for this and couldn’t see me for that. I don’t honestly think she poured her heart and soul into it” (Female, ACE Participant, Data source C)

### Peer volunteering challenges

Clarity of their own motives for volunteering helps volunteers avoid frustration and disappointment. As one volunteer stressed, being available per se is not a good enough reason for volunteering if it is not based on more internalised motives.

“I probably could have started volunteering for the wrong reasons; because I had spare time. But if there’s a purpose behind it, that’s when it works” (Male, Older Volunteer, Data source B)

Peer volunteers recounted the complexity of managing participant relationships and expectations. These ranged from caution regarding a participant’s possible overdependence on a volunteer’s support to the volunteer not feeling needed or required. Coping with these diverse experiences was a concern.

“I was in a bit of a quandary; it was obvious that my participant was having problems with his health. I didn’t know whether I should volunteer to go round … I didn’t want to become a sort of health visitor, or carer” (Male, ACE Activator, Data Source B)

“I would offer to take her out, but I think if I started that, I don’t know where it’s going to lead. So, I have been a little bit careful, she wanted me to go there on a Saturday, and I thought no, I’ve had to push it back to a Friday” (Female, Older Volunteer, Data source B)

Another Activator reported a puzzling situation where the participant having signed-up, seemed to begrudge engaging with them.

“I did feel at one time that she resented me being there …. she felt that I was her minder or something” (Female, ACE Activator, Data Source B)

Other challenges reported by ACE Activators included participants’ health issues, time constraints and difficulties in engaging and supporting participants not motivated to initiate behaviour change.

“She didn’t want to do anything. … And I sort of said, “Well, I don’t know what you’re doing on this programme, you know. Tell me what you do want to do” (Female, ACE Activator, Data source B)

Some challenges with transport to sessions were also raised, particularly in rural areas where public transport was irregular and limited.

“… in villages it is more difficult in the area I live because there isn’t any public transport. … so, there’s much more reliance on volunteer drivers” (Female, ACE Activator, Data source B)

### Peer volunteer training needs

The majority of activators found ACE training simple, well-tailored and supported the ACE programme delivery requirements. However, some activators found it rather time-consuming and somewhat academic in tone.

“Certainly, the training I felt was too long, two days, too academic, it just wasn’t right for some of the volunteers” (Female, ACE Coordinator, Data source A)

Most managers suggested that encouraging volunteers’ participation requires a simple, short, tailored approach to the joining process and training programme. Important elements of the training included strategies to deal with challenges, manage relationships with participants and foster collegiality.

“I felt a bit isolated; I didn’t want to go round knocking on his door, it was difficult. But the training guided me in how I should approach him” (ACE Activator, Data source B)

The ACE Activators felt the training programme should be flexible and support volunteers to apply their practical skills and knowledge appropriately, in addition to new skills acquired from the training. They also highlighted the importance of focussing on convenience, frequency, provision of feedback and use of incentives when planning training sessions ensuring that advance notice of training dates is given at the time of recruitment to enhance uptake:

“So, you’ve got that balancing act between guiding people and helping them, and not overwhelming them with too much information” (Female, ACE Activator, Data source B)

“… at the beginning of the programme it would have been useful to have dates for training in our diaries so that we could have kept those dates free” (Female, ACE activator, Data source B)

Providing volunteers with certificates for attending the training programme was very well-received.

“...it’s a recognised training. Something that they feel proud they’ve actually achieved, although it’s not an onerous training it’s practical as well, and at the end of that day they all go away feeling good...so it’s a booster” (Female, Manager, Data source A)

### Recruitment of peer-volunteers

Successful mobilisation of older adults requires the use of diverse recruitment and advertising routes including media, existing volunteering groups and charities to create awareness and promote initiatives to targeted groups of older adults.

“Personal contacts, going talking to people, talking to groups, building relationships with people, seeing them two or three times in seven days. Working closely with partners who are embedded in the community and getting them to buy in” (Female, Community Initiative Manager 2, Data source A).

“I also went to every group to see what was happening; all the singing, bowling and knitting groups where people were already active … and thought, they would be my ideal volunteer” (Female, Community Initiative Manager 2, Data source A).

“You could get something in the Evening Post … .. You could get people that way. We did get people through campaigns in newspapers” (Male, Older volunteer, Data source B).

Other, more individual approaches may allow recruitment of those who are not easy to reach with standard approaches. These could include face to face recruitment helping to overcome the barrier of a “paper heavy” approach, especially for volunteers with limited literacy.

“You’ve got literacy challenges ...the application form is very paper heavy. I understand that people need to read the information sheet. That maybe could come at a later stage. I think really the best contact … is just that initial face to face and chatting” (Female, Community Initiative Manager 2, Data source A).

Recruitment and retention of peer volunteers requires a flexible approach, clarity of roles and realistic time commitment and expectations for all parties involved while maintaining regular communication.

“The second time we did the training, I did ask on the form for 2 years of commitment otherwise we would ask them to pay some of the training costs back. I think that they were more aware that if they committed to it, it was going to be a commitment for a couple of years (Female, UK Charity Manager 1, Data source A).

“I’d say the thing that can be completely underestimated when managing volunteers is the amount of time that goes into coordinating them. You’re constantly having to reassess things and think about what can be changed, or moving planned sessions” (Female, UK Charity Manager 2, Data source A).

The use of targeted questions and references during the screening process are useful strategies to assess volunteers’ suitability for certain roles.

“We’ve got an application form, and I’ll talk or have an email chat with somebody. You can usually make it clear what the commitment is, and then we will do Disclosure and Barring Service check as well. Then either the coordinator of a group or myself would meet the person. You can tell a lot from meeting volunteers if they’ve got through the first two stages” (Female, Manager, Volunteering Service Provider, Data source A).

Developing an evidence base that allows the peer-volunteering initiative to evolve and adapt as a “real life” programme requires feedback from all stakeholders involved and robust evaluation of its effectiveness. Testing new models in comprehensive research studies provides new initiatives with the time and space to obtain a brand name. That is key to a successful transition to large-scale implementation and recruitment of volunteers.

“I think it will work better when it’s no longer an academic study...that adds more paperwork. There is just that thing about allowing it to build momentum, to become something that people are aware of, not just a new idea. People don’t like doing something new. They want to know somebody who’s done it” (Female, Community Initiative Manager, Data source A).

### Retention of peer-volunteers

#### Administrative support

A significant aspect of volunteering initiatives that is easily overlooked is providing sufficient administrative support ensuring the best use of volunteers’ time.

“We now have a charity log system to manage volunteers, clients and everything. …. because obviously for reporting it makes life a lot easier. But it puts another onus on the volunteers. We have had a few drop off because, the paper work got a bit too much; whereas before they would you know keep their own register in a file, but because of the data management legislation we have to change that …. They are not allowed to keep all the data in a file with them” (Female, UK Charity Manager, Data source A).

“You know volunteers can only do so much I think, but you do need to have this … it’s a sort of impetus behind the scenes that just keeps it going (Female, Older volunteer, Data source B).

“I think things do work best when there is a designated coordinator. It is really important I think to have a dedicated volunteer management type role” (Female, UK Charity Manager 2, Data Source A).

#### Peer support network

The importance of providing peer volunteers with a strong support network to maintain contact, facilitate sharing of ideas, achievements, challenges, and provide feedback and advice was stressed by both volunteers and managers.

“I found it useful to find out, there were some participants that were like mine, that other people had got those problems, so I thought ‘oh it wasn’t so bad after all’, it was a general problem type of thing” (Female, ACE Activator, Data source B).

ACE Activators provided largely positive feedback on the on-going volunteer support events but highlighted the need for these to be scheduled well in advance to ensure high levels of attendance. However, such events need careful management to avoid any sense of competitiveness and volunteers feeling judged.

“I think that would set up, ‘is this becoming competitive?’ or ‘am I being judged for the way I’m, working?” (Male, Older Volunteer, Data source B).

Managers of other volunteering initiatives did find that over time, engaging established volunteers in top-up or refresher training sessions could be challenging.

“We put on all sorts of wonderful training and think people will be really interested. But actually, encouraging volunteers to come along to that training, the take up is quite bad (Female, Manager, Data source A).

#### Clarity of the volunteering role

Managers highlighted that professionals who manage volunteers need to be mindful that volunteering is not an ordinary job and manage their own expectations accordingly.

“You can’t tie them in … you’ve got to be careful about the legal issues around making anything look like a job as opposed to a volunteer role” (Female, Manager, Volunteering Service Provider, Data source A).

#### Matching peers

Shared interests and geographical proximity were considered important elements for matching peers.

“if you match two people that were police officers, … or you could have two people who have lived abroad. Perhaps it’s about finding common interests” (Female, UK Charity Manager 2, Data source A).

“I felt that I didn’t like to have my friend (peer volunteer), … come more often than was necessary because she was some distance away” (Female, ACE Participant, Data source C).

In ACE, participants and peer-volunteers were asked for basic information about their interests. Based on this and researchers’ assessment of their personal characteristics, peers were matched. For example, dog owners were successfully paired, as were those with a previous interest in dancing and those who enjoyed live music. Some pairings were not successful, so an optional no-blame break-point after a trial period is a precaution to consider.

“Don’t be afraid if it doesn’t work out because it’s very difficult to match. … we tend to do it on a trial basis really” (Female, ACE Activator, Data source B).

#### Funding of expenses and activities

Managers of volunteering programmes emphasised the importance of a budget for covering volunteers’ expenses, particularly to avoid excluding potential volunteers from low-income groups.

“You’re going to need to make sure you’ve got money set aside to reimburse those expenses. You wouldn’t want anyone to be out of pocket as a result of giving up their time for you” (Female, UK Charity Manager 2, Data source A).

In addition, resources should ideally be available to fund a reward scheme/recognition for engagement and effective volunteering, in the form of certificates, events and small tokens of appreciation.

“And volunteer thank you events so they feel recognised and supported” (Female, ACE Activator, Data Source B).

#### Building long-term partnerships

Recruiting and training volunteers is costly in terms of funding and resources. Good communication, effective support, (optional) top-up training, thank you/social events (see above), and strong volunteer networks are important strategies to promote long-term volunteer retention.

“My retired volunteers … have often worked for us for years and years” (Male, ACE Activator, Data source B).

“I try and partner volunteers up and get them working together in a little hub …. just to try and strengthen the bond between the network of volunteers” (Female, UK Charity Manager 1, Data source A).

Key strategies for retaining volunteers include clarity of the role and time commitment, clear goals and expectations from all parties, flexibility of delivery and maintenance of clear communication at all stages.

“Make it really clear. 6 months, have that end goal” (Female, Manager, Volunteering Service Provider, Data source A).

“Having task descriptions or role descriptions is quite helpful” (Female, Manager, Volunteering Service Provider, Data source A).

“I think it’s really about being just really open and transparent about what the volunteering would entail” (Female, ACE Activator, Data source B).

## Discussion

Drawing from the experience of a range of stakeholders, the findings confirm the value of peer volunteering in community initiatives promoting active ageing [12, 13, 29]. Peer volunteers reported a range of benefits resulting from engagement with their roles including increases in psychological (personal fulfilment), social (opportunities to reconnect with the local community) and physical wellbeing (more active engagement in local initiatives). A strong sense of altruism, a genuine wish to support other people and the need for a new purpose and structure in everyday life due to changes in life circumstances (bereavement, retirement) were key motives reported in our study. Similar findings were also highlighted in recently published literature [34, 35].

The role of a peer volunteer is demanding. Comments from volunteers indicate that being effective in the role requires high levels of commitment and reliability, a good sense of humour, interpersonal skills, and the ability to relate with people from all walks of life. In our study, participants stressed that the ACE programme, and other active ageing initiatives based on dyadic approaches of peer volunteering, would benefit from pairing volunteers and recipients based on their geographical proximity and common interests (e.g. love for music, having a dog). The importance of considered pairing is supported by literature highlighting the effectiveness of peer volunteers at promoting active ageing when paired successfully with their inactive peers [36, 37]. A recently published systematic review and meta-analysis of 59 trials of dyadic physical activity interventions also highlighted that peer dyadic approaches and having shared target-oriented goals were associated with larger effect sizes [38].

Recruiting volunteers appropriate to the role is a key factor for success for peer volunteering initiatives. A one size fits all approach will not work with such initiatives as the personal characteristics of volunteers and the contexts in which they operate vary considerably. Participants in our study stressed that a clear role description and a detailed breakdown of the level of commitment and the expectations for the role drives a successful recruitment strategy. They highlighted that peer volunteers in active ageing initiatives may face their own challenges (e.g. being the carer for a family member, loss of significant other, part-time work commitments, recovering from a health condition). This finding is supported by a recently published review of community contributions in later life focussing on ways to attract and retain a more diverse pool of peer volunteers [39]. These include age-friendly, flexible and inclusive initiatives focussing on widening the pool of volunteers and supporting the continuing contribution of older volunteers, especially after changes in their personal circumstances.

Participants in this study stressed that the volunteering nature of initiatives, even though it is usually unpaid, does not equate to an amateur or an ad hoc approach. On the contrary, based on volunteer responses, we would say that because of the lack of financial reward these initiatives need to be very efficiently organised with all activities time-tabled well in advance to allow volunteers sufficient time to diarise all requested commitments. Therefore, planning and operating such initiatives needs the support of a responsive administrative team with a designated volunteer manager or coordinator.

Training and on-going support are important to retaining volunteers. The delivery of effective training is not an easy task as volunteers’ educational background differ significantly and some degree of individual tailoring may be required. Participants in our study suggested short, comprehensive training events, inclusive of all abilities, which support the needs of the particular initiative and specific roles within it. A key factor for retaining volunteers is the development of volunteering networks where volunteers support each other through empathy, sharing stories and strategies. Managers who support and allow autonomy in decision-making are favoured. Similarly, Vareilles et al. [40], proposed that for successful mobilisation of community health volunteers, clear roles, provision of skill-based and on-going training, incentives, supervision and logistical support for task distribution and implementation need to be firmly incorporated in the programme design.

Our study showed that managers and coordinators responsible for recruiting volunteers to peer led initiatives value having a comprehensive understanding of the motives of potential volunteers. This understanding enables them to provide the appropriate support to increase adherence and avoid drop-outs and negative experiences associated with participation in the programme. They stressed that the motives for volunteering are likely to change with experience, need to be regularly re-assessed and support adjusted accordingly [41].

In dyadic peer volunteering initiatives, like ACE, volunteers may face the added burden of being paired with participants who do not fully engage with the initiative and that may be demoralising for the volunteer. Training on how to deal with participants’ lack of motivation, manage peer relationships and expectations from participants are key for supporting volunteers and retaining them in the programme. Allen et al. [42], stressed that the long-term impact of volunteering on peers’ physical, cognitive, and emotional functioning should be considered. Participants in this study reported the importance of being very clear and strict about the limits of the volunteer commitment and of communicating this to both volunteers and participants to avoid creating false expectations and dependencies. Not doing that can be emotionally draining for volunteers and negatively impact retention [43].

Finally, an inclusive approach where multiple stakeholders contribute towards the planning, creation, implementation and evaluation of peer-volunteering initiatives was reported as the only way to ensure the successful delivery of peer volunteering initiatives. Participants in our study stressed that having a holistic understanding of the programme and the key players involved enabled them to appreciate the bigger picture and execute their roles successfully. These findings support the focus of public health strategy on mobilising individuals as community assets within whole systems of influence [1]. Evaluation of these systems-oriented approaches needs to go beyond the individual level and consider the role of family, community and population-level systems in motivating and sustaining health behaviour change [44, 45].

The study supports the current focus on mobilising individuals as effective community assets empowering inactive and disengaged older people to re-engage with their communities [1]. A list of 23 actions improving the process of recruitment, implementation and retention of peer volunteering schemes is presented in Table 2.

**Table 2:** Actions for three key stages of a peer volunteering programme

Set up	
▪ Acquire sufficient funding for volunteer management/coordination, volunteer training, expenses and incentives prior to project commencement	
▪ Set realistic volunteer recruitment targets and timescales	
▪ Design and implement a programme evaluation. Work with the funder to ensure this will deliver the outcome data they require	
Recruitment	
▪ Use appropriate, diverse advertising and recruitment and joining routes focusing on delivering a diverse volunteer cohort	
▪ Where appropriate target those whose life circumstances are changing i.e. retirement, moving to a new area or end/reduction of (grand) childcare responsibilities	
▪ Incorporate motives for volunteering into recruitment campaigns including positive outcomes for recipients (altruism), opportunity for volunteer to (re) connect with their community and personal fulfilment	
▪ Design roles to deliver popular benefits of volunteering i.e. personal fulfilment, purpose, acquisition of new skills, increased social and community connections	
▪ Provide a clear volunteer role description	
▪ Provide accurate details of time commitment and period of engagement	
▪ Interview potential volunteers for characteristics that match well with the role including a good sense of humour, good interpersonal skills and the ability to relate to peers	
Implementation and Retention	
▪ Request that all volunteers claim expenses so that no-one feels they cannot afford to volunteer	
▪ Develop and deliver a simple, clear training programme providing the knowledge and skills required for the specific volunteering role	
▪ Prioritise shared interests and geographical proximity when pairing older adults	
▪ Build in a break-point when both volunteer and participant can request a no-blame change of pairing partner	
▪ Provide regular feedback, recognition and incentives to support volunteer retention	
▪ Engage volunteers in discussing their preferences and needs in terms of on-going/ top-up training	
▪ Initiate and support peer volunteer support networks	
▪ Develop and maintain a structure for good, on-going, two-way communication	
▪ Ensure sufficient professional volunteer management/coordination resource is available	
▪ Build resilience through a sufficient bank of trained volunteers to cope with absences and holidays	
▪ Work on developing a pathway to sustaining the programme	
▪ Use outcome data to report impact to funders and support programme roll out and applications to additional funding streams	
▪ Initiate partnerships with community stakeholders	

### Strengths and limitations

Evaluation of dyadic peer volunteering schemes targeting older adults in particular is scarce [38]. The diverse perspectives captured in this study, ranging from peer volunteers, recipients of dyadic peer volunteering actions, volunteer managers and volunteering service providers allowed an in-depth understanding of motives, perceived benefits, characteristics, challenges, training requirements, recruitment strategies and ways to develop a sustainable model for delivering dyadic peer-volunteering active ageing schemes. These perspectives however, reflect experiences of participants living and operating in one city expressing the challenges faced within the specific context of their initiatives. The findings of this study focus on peer volunteering initiatives defined with age as the key common characteristic. Defining peer as ‘common characteristics other than age’ requires further research and the findings of this study may not generalise to those approaches.

Volunteers are more likely to be female with high levels of community involvement and established wider support from friends and neighbours [46, 47]. Out of the 13 ACE Activators interviewed, 10 were women. Male volunteers’ experiences and challenges may differ and we need studies that capture more of their voices.

Finally, the policy framework and practical considerations for the organisation of such initiatives may be different outside a UK context. These differences need to be taken into consideration when comparing the findings of this study with studies examining peer volunteering initiatives outside the UK.

## Conclusions

The voluntary sector is ideally placed to deliver low-cost and effective interventions and to increase access to disadvantaged populations [14, 43]. However, there have been few high quality trials evaluating community approaches that mobilise peer volunteers to promote active ageing [36]. The findings of this study provide a useful framework of actions on how to optimise the contribution of peer-volunteers, particularly to dyadic active ageing initiatives. These include tailored recruitment routes, clarity of role and time commitment, assigning clear goals and expectations for all parties involved, provision of an on-going support network and feedback and recognition of effort, a dedicated team to build resilience, provide administrative support and monitor all aspects of the initiative and good channels of communications across all stakeholders. Future studies will need to evaluate the success of implementation and the long-term maintenance of dyadic peer volunteering active ageing initiatives and their impact on health and wellbeing.

## Supplementary Information

**Additional file 1.** Motives, benefits of volunteering, skills and characteristics of a good peer-volunteer: Qualitative data.

**Additional file 2.** Peer volunteering challenges and training needs: Qualitative data.

**Additional file 3.** Recruitment and retention of peer volunteers: Qualitative data.

**Additional file 4.** Proposed strategies for designing peer volunteering initiatives: Qualitative data.

## Data Availability

The datasets used and or analysed during the current study are available from the corresponding author on reasonable request.

## Abbreviations

ACE: Active, Connected, Engaged programme
COREQ: Consolidated criteria for reporting qualitative research
