# Supplementary material for: Mobilising people as assets for active ageing promotion: a multi-stakeholder perspective on peer volunteering initiatives
Source: BMC Public Health. 2021 Jan 18;21:150. doi: 10.1186/s12889-020-10136-2 (PMC7812118; doi:10.1186/s12889-020-10136-2)
Supplement: Supplementary file 1 — Additional file 1. Motives, benefits of volunteering, skills and characteristics of a good peer-volunteer: Qualitative data. [file 12889_2020_10136_MOESM1_ESM.docx]

**Additional file 1.** **Motives, benefits of volunteering, skills and characteristics of a good peer- volunteer**

| **Themes and subthemes** | **Sample Quotes** |
| --- | --- |
| **Motives for peer volunteering** | |
| - Altruism - Changes in life circumstances (retirement, bereavement, house move) - Opportunities to reconnect with the community - Personal fulfilment | *“I came across Calling Time because one of the heart-breaking adverts that a UK charity put out at Christmas about loneliness. The thought of my grandma having no one to talk to really upset me, so you know the thought of someone else’s grandma out there who doesn’t have no one to talk to”; Female, UK Charity Manager 2, Data source A*  *“I lived in another part of town... I was re-housed up here. And I’d just taken early retirement so... I was looking for something to do. I came in to see what was going on I think... but uh, I got well roped in”; Male, Older Volunteer, Data source B*  *“It’s nice having a new social circle; I’ve got to meet other people I wouldn’t have come across”; Male, ACE Activator, Data source B*  *“He’s so grateful you know and that makes me feel really good, I’ve done something good today. I made an old man happy”; Female, 65 years, Older Volunteer, Data source B* |
| **Benefits of peer volunteering** | |
| - Personal fulfilment (feeling valued and appreciated) - Provision of a purpose - Acquisition of new skills and knowledge - Increased social connections - Awareness of interesting community activities - Reason to go out more/ increase physical activity - Increased confidence for interacting with peers and engaging in other activities - Opportunity to identify related needs of some older isolated adults and their families | *“it’s just understanding and appreciating how important, kind of the importance that some of the older people place on the volunteers. They really do mean a lot to them you know; they really are quite treasured; Female, UK Charity Manager 2, Data source A*  *“I definitely think they’ve made new friends and it’s given them a bit of a purpose in life. I think it’s a tw-way thing, they’re giving, but they’re also getting quite a lot back from it”; Female, UK Charity Manager 1, Data source A*  *“Being an organiser, it has an effect. Because you’re doing a lot of research, my search for ideas, my search for money, my search for a bit of inspiration. It’s broadened my knowledge tremendously; it’s broadened most of us”; Male, Older Volunteer, Data source B*  *“I feel I have friends and I can go places instead of, oh, sitting in here all the time”; ACE Participant, Data source C*  *“For me it's the reintegration into a social situation. It gives people a purpose. Once people are integrated … you know, you're expected somewhere and wanted somewhere”; Female, ACE Study Phase 3 Manager, Data source A*  *“She introduced me to a lot of things that I would like to have done but would never have known about really”; ACE Participant, Data source C*  *“It’s nice having a new social circle, I’ve got to meet other people… I wouldn’t have come across”; Male, ACE Activator, Data source B*  *“Oh, just individual stories of people who are very isolated, very low in confidence. Really low mood, bordering on mental health issues and how their lives have turned around”; Female, ACE Study Phase 3 Manager 1, Data source A*  *“Once we chatted and we went off to line dancing, she didn’t look back. I mean, she doesn’t need me now, at all. She’s found her own network and it’s given her the confidence to go out there”; Female, ACE Activator, Data source B*  *“He's just like I love it, it's likely he wouldn't have the confidence to do that before his volunteer. He lives with his wife and it's really changed her life”; Female, ACE Study Phase 3 Manager 2, Data source A*  *“It is quite likely that if somebody is socially isolated there might be something else going on as well. A participant had no way of keeping the milk cold, weren't eating healthily so were getting more and more overweight which wasn't helping their moods, making them less likely to go out. Through our grant giving, we were able to give him a fridge freezer”; Female, ACE Study Phase 3 Manager 1, Data source A* |
| **What makes a good peer-volunteer?** | |
| - Committed and reliable - Good sense of humour - Friendly and approachable disposition - Ability to relate with peers - Ability to motivate older people - Clarity of communication - Shared interests | *“And then she [peer volunteer] informed me she was away for x number of weeks and couldn’t see me for this and couldn’t see me for that. I don’t honestly think she poured her heart and soul into it and she had so many other commitments”; Female, ACE Participant, Data source C*  *“I can honestly say, never once since I met her, has she let me down”; Female, ACE Participant, Data source C*  *“I just make people laugh and things, because I think that’s the way forward really. I want them to feel relaxed”; Female, ACE Activator, Data Source B*  *“I found her easy to talk to and I didn’t know what to expect but I found her very friendly”; Female, ACE Participant, Data source C*  *“What’s critical are the rapport building skills of the people you have coordinating, that is critical in motivating volunteers if you get people who can… make those people feel valuable it will overcome a lot of hurdles; Female, ACE Coordinator, Data source A*  *“The biggest thing I found was motivation. It was motivating people – to do things, trying things out, some work, some don’t”; Female, ACE Activator, Data Source B*  *“To be able to communicate easily is the main thing and to be able to encourage...”; Female, ACE Activator, Data Source B*  *“And also, I thought, well everybody walks, it’s my sort of thing, being in a walking group, but she didn’t like walking, so we didn’t really get on greatly anyway”; Female, ACE Activator, Data source B*  *“We’re just seeing about whether having more aligned interests and backgrounds, you know has a greater impact; if you match two people that were police officers, …or you could have two people who have lived abroad for a number of years. Perhaps it’s about finding common interests”; Female, UK Charity Manager 2, Data source A* |
