# Supplementary material for: Mobilising people as assets for active ageing promotion: a multi-stakeholder perspective on peer volunteering initiatives
Source: BMC Public Health. 2021 Jan 18;21:150. doi: 10.1186/s12889-020-10136-2 (PMC7812118; doi:10.1186/s12889-020-10136-2)
Supplement: Supplementary file 2 — Additional file 2. Peer volunteering challenges and training needs: Qualitative data. [file 12889_2020_10136_MOESM2_ESM.docx]

**Additional file 2. Peer volunteering challenges and training needs**

| **Themes and subthemes** | **Sample Quotes** |
| --- | --- |
| **Peer volunteering challenges** | |
| - Volunteering for the wrong reasons - Managing volunteer-participant relationships; participant overdependence - Time constraints - Feeling redundant due to participant becoming independent - Reluctance to enrol/ slow take-up by older people - Transportation issues - Participant health issues - Lack of motivation in older adults | *“I probably could have started volunteering for the wrong reasons; because I had spare time. But if there’s a purpose behind it that’s when it works”; Male, Older Volunteer, Data source B*  *“I think it’s really about being just really open and transparent about what the volunteering would entail. Because I think it helps having a transparent understanding of what’s actually involved”; Female, ACE Activator, Data source B*  *“I would offer to take her out, but I think if I started that, I don’t know where it’s going to lead. So I have been a little bit careful, she wanted me to go there on a Saturday, and I thought no, I’ve had to push it back to a Friday”; Female, 65 years, Older Volunteer, Data source B*  *“I felt redundant because she seemed quite independent, you know, and was already doing things”; Female, ACE Activator, Data Source B*  *“The thought of having a stranger, somebody they don't know coming into their home and talking to them. It can just be a bit too much for some people. But people have to hear about these things a lot and then if they know somebody who's done it and these things do just take time to build”; Female, ACE Study Phase 3 Manager 1, Data source A*  *“…in villages it’s more difficult in the area I live because there isn’t any public transport. I think there’s one bus a day if you’re lucky. So, there’s a much more reliance on volunteer drivers”; Female, ACE Activator, Data source B*  *“I was in a bit of a quandary; it was obvious that my participant was having problems with his health. I didn’t know whether I should volunteer to go round, but on the other hand I didn’t want to become a sort of health visitor, or carer”; Male, ACE Activator, Data Source B*  *“She didn’t want to do anything. I was like, “Oh!” And I sort of said, “Well, I don’t know what you’re doing on this programme, you know. Tell me what you do want to do”; Female, ACE Activator, Data source B* |
| **Peer-volunteer training needs** | |
| - Simple and short; training and joining requirements e.g. Disclosure & Barring Service (DBS) checks off putting - Tailored to specific role/ project - Impart knowledge and skills to manage relationships and challenges - Incorporate volunteer support systems; supportive and not instigate competition among volunteers - Flexible to enable appropriate application of own knowledge and skills - Session planning; communicate dates, include incentives (social events), provision of feedback and some form of recognition | *“The data checks and DBS checks; I know, I know it puts a lot of volunteers off really”; Female, UK Charity Manager 1, Data source A*  *“Certainly, the training I felt was too long, two days, too academic, it just wasn’t right for some of the volunteers; Female, ACE Coordinator, Data source A*  *“So you’ve got that balancing act between guiding people and helping them, and not overwhelming them with too much information”; Female, ACE Activator, Data source B*  *“I mean if we’re going to be asking them to download apps and what have you, then we’re gonna have to provide them with the equipment to do it and the training, so that they know what they’re doing”; Female, UK Charity Manager 1, Data source A*  *“I felt a bit isolated; I didn’t want to go round knocking on his door, it was difficult. But the training guided me in how I should approach him”; ACE Activator, Data source B*  *“I think that would set up, ‘is this becoming competitive?’ or ‘am I being judged for the way I’m, working?’ And I think they’ll all find success, and having found success they’ll have the confidence to share it, but I wouldn’t set up a network immediately”; Male, Older Volunteer, Data source B*  *“I thought it was useful guidelines. In the end you do it, you do it your own way”; ACE Activator, Data source B*  *“…the beginning of the programme it would have been useful to have dates in our diaries so that we could have kept those dates free“; Female, ACE Activator, Data source B*  *“..it’s a recognised training. Something that they feel proud they’ve actually achieved, although it’s not an onerous training it’s practical as well, and at the end of that day they all go away feeling good...so it’s a booster”; Female, Manager, Data source A*  *“We put on all sorts of wonderful training and think people will be really interested. It’s very relevant to what they do and they’ve asked us about how to deal with X, Y, Z so let’s put the training on. But actually, encouraging volunteers to come along to that training, the take up is quite bad; Female, Manager, Data source A* |
