# Supplementary material for: Mobilising people as assets for active ageing promotion: a multi-stakeholder perspective on peer volunteering initiatives
Source: BMC Public Health. 2021 Jan 18;21:150. doi: 10.1186/s12889-020-10136-2 (PMC7812118; doi:10.1186/s12889-020-10136-2)
Supplement: Supplementary file 3 — Additional file 3. Recruitment and retention of peer volunteers: Qualitative data. [file 12889_2020_10136_MOESM3_ESM.docx]

**Additional file 3. Recruitment and retention of peer volunteers**

| **Themes and subthemes** | **Sample Quotes** |
| --- | --- |
| **Recruitment and retention of peer-volunteers** | |
| - Provision of peer support networks - Acknowledge volunteering role as it is, not a job - Clarify role and time commitment - Prioritise shared interests and geographical proximity when matching peers - Allocate budget for activities - Integrate a personal reward scheme and recognition for volunteers - Maintain good communication strategies - Partnership building | *“I would say, that I would wait a couple of months until they have found their way and then offer them an invitation for networking… then say, would you like to get together?”; Male, Older Volunteer, Data source B*  *“I found it useful to find out, there were some participants that were like mine, that other people had got those problems, so I thought ‘oh it wasn’t so bad after all’, it was a general problem type of thing”; Female, ACE Activator, Data source B*  *“You can’t tie them in. If it becomes like a contract of employment you’ve got to be careful about the legal issues around making anything look like a job as opposed to a volunteer role”; Female, Manager, Volunteering Service Provider, Data source A*  *“Having task descriptions or role descriptions is quite helpful”; Female, Manager, Volunteering Service Provider, Data source A*  *“Make it really clear. 6 months, have that end goal”; Female, Manager, Volunteering Service Provider, Data source A*  *“….we’re just seeing about whether having more aligned interests and backgrounds, you know has a greater impact……… Perhaps it’s about finding common interests”; Female, UK Charity Manager 2, Data source A*  *“I felt that I didn’t like to have my friend (peer volunteer), …come more often than was necessary because she was some distance away”; Female, ACE Participant, Data source C*  *“The other thing from sort of a practical point of view ..obviously volunteers are not paid. You’re going to need to make sure you’ve got money set aside to reimburse (their) expenses. You wouldn’t want anyone to be out of pocket as a result of giving up their time for you”; Female, UK Charity Manager 2, Data source A*  *“And volunteer thank you events so they feel recognised and supported”; Female, ACE Activator, Data Source B*  *“My retired volunteers …have often worked for us for years and years”; Male, ACE Activator, Data source B*  *“I try and partner volunteers up and get them working together in a little hub…. just to try and strengthen the bond between the network of volunteers”; Female, UK Charity Manager 1, Data source A*  *“Recruiting through Neighbourhood Partnerships (charities, societies and clubs).. they have forums and monthly meetings, and activators in the community go so if you can persuade them, they can start to spread the message. Working through volunteer agencies people will come for a bit, they don’t have that sense of ownership over the scheme.”; Manager, Volunteering Service Provider, Data source A* |
