# Supplementary material for: Mobilising people as assets for active ageing promotion: a multi-stakeholder perspective on peer volunteering initiatives
Source: BMC Public Health. 2021 Jan 18;21:150. doi: 10.1186/s12889-020-10136-2 (PMC7812118; doi:10.1186/s12889-020-10136-2)
Supplement: Supplementary file 4 — Additional file 4. Proposed strategies for designing peer volunteering initiatives: Qualitative data. [file 12889_2020_10136_MOESM4_ESM.docx]

**Additional file 4. Proposed strategies for designing peer volunteering initiatives**

| **Themes and subthemes** | **Sample Quotes** |
| --- | --- |
| **Proposed strategies for designing peer volunteering initiatives** | |
| - Use of appropriate, diverse advertising and recruitment routes e.g. media, existing volunteering groups and charities      - Explore diverse joining routes for keen individuals e.g. volunteers with literacy challenges - Use right terminology to promote initiatives to older adults - Set realistic recruitment targets; mindful of roles & time commitment - Ask targeted questions during screening; use of references to assess volunteers’ suitability - Develop a pathway to sustaining the programme - Build resilience - Administrative support-paid staff - Generate evidence using research; support funding applications & build networks necessary for uptake as a community project | *“I think we need to look at different recruitment techniques specific to the area. I think because this particular project was with the University, for some people that's really attractive…. but for some people that's off putting because ... that's just not something that they would necessarily like see themselves as getting involved with”; Female, ACE Study Phase 3 Manager 2, Data source A*  *“You've got literacy challenges ...the application form is very paper heavy. I understand that people need to read the information sheet, that maybe could come at a later stage. I think really the best contact… is just that initial face to face and chatting”; Female, ACE Study Phase 3 Manager 2, Data source A*  *“it’s not a charity, it’s a help group... set up so people can help each other; people helping people”; Male, Older volunteer, Data source B*  *“The second time we did the training, I did ask on the form for 2 years of commitment otherwise we would ask them to pay some of the training costs back. I think that they were more aware that if they committed to it, it was going to be a commitment for a couple of years”; Female, UK Charity Manager 1, Data source A*  *“I’d say the thing that can be completely underestimated when managing volunteers is the amount of time that goes into coordinating them. You’re constantly having to reassess things and think about what can be changed, or can we move that day”; Female, UK Charity Manager 2, Data source A*  *“We’ve got an application form, and I’ll talk or have an email chat with somebody. You can usually make it clear what the commitment is and then we’ll do DBS as well. Then either the coordinator of a group, or myself would meet the person as well. You can tell a lot from meeting volunteers if they’ve got through the first two stages”; Female, Manager, Volunteering Service Provider, Data source A*  *“You know volunteers can only do so much I think, but you do need to have this…it’s a sort of impetus behind the scenes that just keeps it going”; Female, Older volunteer, Data source B*  *“I think things do work best when there is a designated coordinator. It is really important I think to have a dedicated volunteer management type role”; Female, UK Charity Manager 2, Data source A*  *“We now have a charity log system to manage volunteers, clients and everything. So now the paperwork does go back to the office, the registration forms and everything so all our clients go on the charity log. Which is great, that bit is good, because obviously for reporting it makes life a lot easier. But it puts another onus on the volunteers. We have had a few drop off because, the paperwork got a bit too much; whereas before they would you know keep their own register in a file, but because of the data management legislation we have to change that introducing the new consent form. They are not allowed to keep all the data in a file with them”; Female, UK Charity Manager 1, Data Source A*  *“I think it will work better when it's no longer an academic study...that adds more paperwork. There is just that thing about allowing it to build momentum...to become something that people are aware of, not just a new idea. People don't like doing something new. They want to know somebody who's done it”; Female, ACE Study Phase 3 Manager 1, Data source A* |
